# Supplementary material for: Identification of two distinct phylogenomic lineages and model strains for the understudied cystic fibrosis lung pathogen Burkholderia multivorans
Source: Microbiology (Reading). 2023 Aug 1;169(8):001366. doi: 10.1099/mic.0.001366 (PMC10482378; doi:10.1099/mic.0.001366)
Supplement: Supplementary material 1 [file mic-169-1366-s001.pdf]

Supplementary Data

Title: Identification of two distinct phylogenomic lineages and model strains for the understudied cystic fibrosis lung pathogen *Burkholderia multivorans*

Authors: Kasia M. Parfitt[1], Angharad E. Green[2,3], Thomas R. Connor[1], Daniel R. Neill[2], and Eshwar Mahenthiralingam[1]

Affiliations:

[1] Cardiff University, Microbiomes, Microbes and Informatics Group, Organisms and Environment Division, School of Biosciences, Cardiff University, CF10 3AX, UK

[2] Department of Clinical Infection, Microbiology and Immunology, Institute of Infection, Veterinary and Ecological Sciences, University of Liverpool, Liverpool, L69 7BE, UK

Current affiliations:

[3] Big Data Institute, Nuffield Department of Population Health, Li Ka Shing Centre for Health Information and Discovery, Old Road Campus, University of Oxford, Oxford OX3 7LF, UK

Supplementary tables

Table S1. Details of the 283 *B. multivorans* genome sequences analysed in this study

Table S2 Quality statistics of the 73 *B. multivorans* draft bacterial genomes sequenced in this study

Table S3. PCR primer sequences designed for all four *B. multivorans* lineage-specific target genes

Table S4. Growth kinetics of the *B. multivorans* strains showing growth rate, lag phase and maximum OD

Table S5. Swimming and swarming motility within the *B. multivorans* strains

Table S6. Colony morphology and EPS production of *B. multivorans*

Supplementary Figures

Figure S1. MLST-gene phylogeny of the 77 genome *B. multivorans* strain panel

Figure S2. Growth parameters of the *B. multivorans* strains (n = 50) at 48 h

Figure S3. Swimming and swarming motility in the *B. multivorans* strain panel after 24 h incubation at 37°C

Figure S4. Comparison of motility between lineage 1 and lineage 2 *B. multivorans* strains

Figure S5. Biofilm formation of the *B. multivorans* strain panel (n = 49) after 24 h

Figure S6. Comparison of biofilm formation between the *B. multivorans* lineages

Table S1. Details of the 283 *B. multivorans* genome sequences analysed in this study

Strain	Isolate Type	Lineage	Accession Number	Sequence Type (ST)	Other name	Origin
701_BMUL	NA	2b	GCF_001058025.1	16		Washington, USA
800_BMUL	NA	2b	GCF_001058485.1	16		Washington, USA
ATCC_17616	ENV	2a	GCF_000018505.1	21		USA
ATCC_BAA-247	CF	1	GCF_000959525.1	650		Brussels, Belgium
AU10047	CF	1	GCF_002980655.1	564		USA
AU10086	CF	2b	GCF_002980675.1	16		USA
AU10398	CF	2a	GCF_002980695.1	1512		USA
AU10897	CF	2b	GCF_002980715.1	1794		USA
AU11204	CF	1	GCA_002980785.1	1803		USA
AU11233	CF	2a	GCF_002980775.1	1800		USA
AU11358	CF	1	GCF_002981015.1	646		USA
AU11772	CF	Other	GCF_002981035.1	179		USA
AU1185	NON	1	GCF_001718755.1	18		USA
AU12481	CF	2a	GCF_002981075.1	1793		USA
AU13919	CF	2b	GCF_002981095.1	1807		USA
AU14328	CF	2b	GCF_002980825.1	1087		USA
AU14364	CF	2a	GCF_002980815.1	418		USA
AU14371	CF	2b	GCF_002980855.1	1792		USA
AU14786	CF	Other	GCF_002980875.1	179		USA
AU15814	CF	2a	GCF_002980895.1	418		USA
AU15954	CF	2b	GCF_002980905.1	190		USA
AU16734	CF	1	GCF_002981615.1	1788		USA
AU17135	CF	1	GCF_002981135.1	1023		USA
AU17534	CF	2a	GCF_002980935.1	880		USA
AU17545	CF	2a	GCF_002980995.1	623		USA
AU18096	CF	2a	GCF_002981145.1	603		USA
AU19518	CF	2a	GCF_002981195.1	630		USA
AU19564	CF	2b	GCF_002981155.1	1796		USA
AU19659	CF	1	GCF_002981255.1	1789		USA
AU19729	CF	2a	GCF_002981215.1	623		USA
AU20929	CF	2b	GCF_002981635.1	715		USA
AU21015	CF	2b	GCF_003048355.1	622		USA
AU21596	CF	2b	GCF_002981675.1	1801		USA
AU21747	CF	2a	GCF_002981315.1	21		USA
AU22436	CF	1	GCF_002981695.1	666		USA
AU22892	CF	2b	GCF_002981295.1	190		USA
AU23365	CF	1	GCF_002981715.1	18		USA
AU23668	CF	2a	GCF_002981725.1	1802		USA
AU23690	CF	1	GCF_002981325.1	1791		USA
AU23919	CF	Other	GCF_002981335.1	179		USA
AU23995	CF	2b	GCF_002981755.1	1790		USA
AU24277	CF	2b	GCF_002981375.1	625		USA
AU25057	CF	1	GCF_002981775.1	1787		USA
AU25543	CF	2a	GCF_002981815.1	603		USA
AU26250	CF	2b	GCF_002981835.1	190		USA
AU27706	CF	1	GCF_002981395.1	18		USA
AU28069	CF	2a	GCF_002981845.1	630		USA
AU28442	CF	1	GCF_002981415.1	645		USA
AU29198	CF	2b	GCF_002981455.1	1795		USA
AU30050	CF	1	GCF_002981485.1	1786		USA
AU30438	CF	1	GCF_002981515.1	1785		USA
AU30441	CF	1	GCF_002981535.1	1383		USA
AU30760	CF	2b	GCF_002981555.1	1797		USA
AU4507	CF	2b	GCF_002981595.1	891		USA
BCC0005	CF	2b	ERS785011	19	LMG 18822; C5393	British Colombia, Canada
BCC0006	CF	1	ERR4672189	650	4F	Toronto, Canada

BCC0008	CF	2b	ERS784904	899	LMG 16660; C1576	Glasgow, UK
BCC0009	CGD	1	ERR4672190	1530	LMG 18824	USA
BCC0010	NON	1	ERS784919	18	LMG 16665; C1962; FC0762	UK
BCC0031	CF	2b	ERS785002	191	KE1; CO514	British Colombia, Canada
BCC0032	CF	2a	ERR4672191	191	C3865; HA1	British Colombia, Canada
BCC0033	CF	2b	ERR4672192	16	C5568	California, USA
BCC0037	CGD	2a	ERS785053	271	CEP0178	California, USA
BCC0043	CF	2b	ERR4672194	806	C1528	Belfast, UK
BCC0047	CF	2a	ERR4672260	1077	C1712	Aberdeen, UK
BCC0050	ENV	2b	ERS785036	439	J0365	UK
BCC0059	CF	2b	ERS785022	287	CEP0494	Montreal, Canada
BCC0065	NON	2b	ERR4672261	16	CEP0600	Oregon, USA
BCC0066	CF	2a	ERR4672262	880	CEP0602	British Colombia, Canada
BCC0067	CF	1	ERS784935	189	CEP0603	Montreal, Canada
BCC0068	CF	2b	ERR4672263	2213	CEP0604	Montreal, Canada
BCC0074	CF	2a	ERR4672264	618	HI-2308	USA
BCC0075	CF	2b	ERR4672265	899	C1579	Glasgow,UK
BCC0079	CF	2b	ERR4672266	1792	C3430	British Colombia, Canada
BCC0080	CF	1	ERR4672267	1964	C4861	British Colombia, Canada
BCC0082	CF	2b	ERR4672268	2220	C6100	British Colombia, Canada
BCC0084	CF	1	ERR10387434	195	C6398	British Colombia, Canada
BCC0087	CF	2b	ERR4672270	199	C6935	British Colombia, Canada
BCC0089	CF	2b	ERS785033	191	C7274	British Colombia, Canada
BCC0093	CF	1	ERZ1756586	1023	C7363	British Colombia, Canada
BCC0096	CF	2b	ERR4672271	305	C7062	British Colombia, Canada
BCC0099	CF	2b	ERS785069	305	C7510	British Colombia, Canada
BCC0101	CF	1	ERR4672272	304	C8298	British Colombia, Canada
BCC0102	CF	2b	ERS784954	190	C8467	British Colombia, Canada
BCC0115	CF	1	ERS784971	195	Patient Q	Washington, USA
BCC0134	CF	2a	ERZ1756605	1088	CEP0699	Denmark
BCC0141	CF	1	ERR4672273	198	CEP0740; AU0071	USA
BCC0149	CF	1	ERS785050	1023	FC0328	USA
BCC0175	CGD	1	ERS785024	355	FC0442	Colorado, USA
BCC0181	CF	2b	ERZ1756585	16	LMG 14273	Belgium
BCC0188	CF	2a	ERR4672274	196	LMG 14276	Belgium
BCC0225	CF	2a	ERR4674034	618	CEP0837	Manitoba, Canada
BCC0241	NON	2b	ERR4672275	605	CEP0935	Minnesota, USA

BCC0244	CF	2a	ERS785004	1088	CEP0938	British Colombia, Canada
BCC0246	CF	2b	ERR4672279	273	NU9366	New Zealand
BCC0247	CF	2b	ERR4674976	16	CEP0949	New Zealand
BCC0255	CF	2b	ERZ1756587	1792	CEP0966	Australia
BCC0264	CF	2a	ERR4676953	274	CEP0992	New South Wales, Australia
BCC0266	CF	2a	ERR4672280	2228	CEP0995	New South Wales, Australia
BCC0269	CF	2b	ERR4672281	2219	CEP1000	New South Wales, Australia
BCC0292	CF	1	ERZ1756588	1786	C1409; CEP0133	Edinburgh, UK
BCC0293	CF	1	ERZ1756589	487	C1406; BCC0293	Oklahoma, USA
BCC0300	CF	2b	ERZ1756590	16	STRAIN 88; CEP0503	France
BCC0303	CF	1	ERR4672282	25	AU0066	USA
BCC0317	ENV	2a	ERR4672283	22	BP102	British Colombia, Canada
BCC0321	ENVH	1	ERZ1756591	2227	C1664; CEP0181	Manchester, UK
BCC0375	CF	1	ERR4672284	117	AU0607	USA
BCC0381	NON	1	ERR4672285	18	LMG 16665	UK
BCC0384	CF	2b	ERR4672589	15	FC0769	British Colombia, Canada
BCC0470	CF	2b	ERZ1756592	1792	MN (#303)	Italy
BCC0493	CF	2b	ERR4672590	806	BELF 1 (#353)	Belfast, UK
BCC0497	CF	2b	ERR4672598	26	BELF 5 (#357)	Belfast, UK
BCC0533	CF	2b	ERZ1756593	19	C3168	British Colombia, Canada
BCC0553	CF	2b	ERZ1756594	16	C6558	British Colombia, Canada
BCC0583	CF	2A	ERZ1756595	21	C9140	British Colombia, Canada
BCC0585	CF	2b	ERZ1756596	199	C8814	British Colombia, Canada
BCC0702	CF	1	ERR4672759	2223	IST453	Portugal
BCC0704	CF	1	ERZ1756597	836	IST455	Portugal
BCC0710	CF	2b	ERR4672760	15	A1-4	Cardiff, UK
BCC0729	CF	2a	ERZ1756598	195	C1628	Cardiff, UK
BCC0737	CF	1	ERR4676914	ND	C1628	UK
BCC0814	CF	1	ERR4674025	180	3-454	Prague, Czech Republic
BCC0865	CF	1	ERR4674026	181	54-1138	Prague, Czech Republic
BCC0901	CF	2b	ERZ1756599	182	90-N320	Prague, Czech Republic
BCC0904	NON	1	ERR4674027	180	93-N331	Prague, Czech Republic
BCC0907	NON	2a	ERZ1756600	630	96-N337	Prague, Czech Republic
BCC0915	CF	1	ERZ1756601	180	104-455	Prague, Czech Republic
BCC0921	CF	1	ERR4674035	181	110-1226	Prague, Czech Republic
BCC0962	CF	1	ERZ1756602	180	151-454	Prague, Czech Republic
BCC0968	CF	1	ERZ1756603	181	157-1226A	Prague, Czech Republic
BCC1147	CF	2b	ERR4674028	305	C9861	British Colombia, Canada
BCC1148	CF	2b	ERR4674030	305	C9862	British Colombia, Canada
BCC1177	CF	1	ERR4674031	195	D0913	British Colombia, Canada
BCC1185	CF	2b	ERR4676921	199	D1348	British Colombia, Canada
BCC1190	CF	1	ERR4674032	18	D1442	British Colombia, Canada
BCC1271	CF	2a	ERZ1756604	21	AU 4543	USA
BCC1272	CF	2a	ERR10387431	21	HI 2229	USA
BCC1367	CF	1	ERZ1756606	25	AU0623	USA

BCC1368	CF	Other	ERR4676903	179	AU1187	USA
BCC1384	CF	1	ERZ1756607	25	AU0508	USA
BCC1385	CF	1	ERR4674033	847	AU4608	USA
BCC1421	CF	2b	ERZ1756608	899	C1576	Glasgow, UK
CF170.0a	CF	2b	GCF_003257435.1	783		Toronto, Canada
CF170.10a	CF	2b	GCA_003256675.1	783		Toronto, Canada
CF170.10b	CF	2b	GCF_003257315.1	783		Toronto, Canada
CF170.10c	CF	2b	GCF_003257325.1	783		Toronto, Canada
CF170.10d	CF	2b	GCF_003258065.1	783		Toronto, Canada
CF170.10e	CF	2b	GCF_003258115.1	783		Toronto, Canada
CF170.10f	CF	2b	GCF_003256685.1	783		Toronto, Canada
CF170.10g	CF	2b	GCF_003256695.1	783		Toronto, Canada
CF170.10h	CF	2b	GCF_003257345.1	783		Toronto, Canada
CF170.10i	CF	2b	GCF_003256725.1	783		Toronto, Canada
CF170.10j	CF	2b	GCF_003258145.1	783		Toronto, Canada
CF170.11a	CF	2b	GCF_003256735.1	783		Toronto, Canada
CF170.11b	CF	2b	GCF_003258135.1	783		Toronto, Canada
CF170.11c	CF	2b	GCF_003256765.1	783		Toronto, Canada
CF170.11d	CF	2b	GCF_003257375.1	783		Toronto, Canada
CF170.11e	CF	2b	GCF_003256805.1	783		Toronto, Canada
CF170.11f	CF	2b	GCF_003258155.1	783		Toronto, Canada
CF170.11g	CF	2b	GCF_003257365.1	783		Toronto, Canada
CF170.11h	CF	2b	GCF_003256775.1	783		Toronto, Canada
CF170.11i	CF	2b	GCF_003257405.1	783		Toronto, Canada
CF170.11j	CF	2b	GCF_003256855.1	783		Toronto, Canada
CF170.1a	CF	2b	GCF_003255945.1	783		Toronto, Canada
CF170.1b	CF	2b	GCF_003257445.1	783		Toronto, Canada
CF170.1c	CF	2b	GCF_003255985.1	783		Toronto, Canada
CF170.1d	CF	2b	GCF_003257475.1	783		Toronto, Canada
CF170.1e	CF	2b	GCF_003256835.1	783		Toronto, Canada
CF170.1f	CF	2b	GCF_003257495.1	783		Toronto, Canada
CF170.1g	CF	2b	GCF_003256865.1	783		Toronto, Canada
CF170.1h	CF	2b	GCF_003255935.1	783		Toronto, Canada
CF170.1i	CF	2b	GCF_003255975.1	783		Toronto, Canada
CF170.1j	CF	2b	GCF_003257515.1	783		Toronto, Canada
CF170.2a	CF	2b	GCF_003256005.1	783		Toronto, Canada
CF170.2b	CF	2b	GCF_003257525.1	783		Toronto, Canada
CF170.2c	CF	2b	GCF_003256015.1	783		Toronto, Canada
CF170.2d	CF	2b	GCF_003256895.1	783		Toronto, Canada
CF170.2e	CF	2b	GCF_003256905.1	783		Toronto, Canada
CF170.2f	CF	2b	GCF_003257545.1	783		Toronto, Canada
CF170.2g	CF	2b	GCF_003256055.1	783		Toronto, Canada
CF170.2h	CF	2b	GCF_003256915.1	783		Toronto, Canada
CF170.2i	CF	2b	GCF_003257575.1	783		Toronto, Canada
CF170.2j	CF	2b	GCF_003256075.1	783		Toronto, Canada
CF170.3a	CF	2b	GCF_003256095.1	783		Toronto, Canada
CF170.3b	CF	2b	GCF_003257595.1	783		Toronto, Canada
CF170.3c	CF	2b	GCF_003257625.1	783		Toronto, Canada
CF170.3d	CF	2b	GCF_003257635.1	783		Toronto, Canada
CF170.3e	CF	2b	GCF_003258535.1	783		Toronto, Canada
CF170.3f	CF	2b	GCF_003257615.1	783		Toronto, Canada
CF170.3g	CF	2b	GCF_003256935.1	783		Toronto, Canada
CF170.3h	CF	2b	GCF_003256105.1	783		Toronto, Canada
CF170.3i	CF	2b	GCF_003257665.1	783		Toronto, Canada
CF170.3j	CF	2b	GCF_003257705.1	783		Toronto, Canada
CF170.4a	CF	2b	GCF_003257685.1	783		Toronto, Canada
CF170.4b	CF	2b	GCF_003256115.1	783		Toronto, Canada
CF170.4c	CF	2b	GCF_003257725.1	783		Toronto, Canada
CF170.4d	CF	2b	GCF_003256985.1	783		Toronto, Canada
CF170.4e	CF	2b	GCF_003256145.1	783		Toronto, Canada
CF170.4f	CF	2b	GCF_003256185.1	783		Toronto, Canada
CF170.4g	CF	2b	GCF_003257005.1	783		Toronto, Canada

CF170.4h	CF	2b	GCF_003256175.1	783	Toronto, Canada
CF170.4i	CF	2b	GCF_003257765.1	783	Toronto, Canada
CF170.4j	CF	2b	GCF_003256225.1	783	Toronto, Canada
CF170.5a	CF	2b	GCF_003256975.1	783	Toronto, Canada
CF170.5b	CF	2b	GCF_003256195.1	783	Toronto, Canada
CF170.5c	CF	2b	GCF_003256995.1	783	Toronto, Canada
CF170.5d	CF	2b	GCF_003257075.1	783	Toronto, Canada
CF170.5e	CF	2b	GCF_003257055.1	783	Toronto, Canada
CF170.5f	CF	2b	GCF_003256255.1	783	Toronto, Canada
CF170.5g	CF	2b	GCF_003256275.1	783	Toronto, Canada
CF170.5h	CF	2b	GCF_003256285.1	783	Toronto, Canada
CF170.5i	CF	2b	GCF_003257085.1	783	Toronto, Canada
CF170.5j	CF	2b	GCF_003256305.1	783	Toronto, Canada
CF170.6a	CF	2b	GCF_003257795.1	783	Toronto, Canada
CF170.6b	CF	2b	GCF_003257755.1	783	Toronto, Canada
CF170.6c	CF	2b	GCF_003257805.1	783	Toronto, Canada
CF170.6d	CF	2b	GCF_003256355.1	783	Toronto, Canada
CF170.6e	CF	2b	GCF_003257135.1	783	Toronto, Canada
CF170.6f	CF	2b	GCF_003257825.1	783	Toronto, Canada
CF170.6g	CF	2b	GCF_003257845.1	783	Toronto, Canada
CF170.6h	CF	2b	GCF_003257095.1	783	Toronto, Canada
CF170.6i	CF	2b	GCF_003256335.1	783	Toronto, Canada
CF170.6j	CF	2b	GCF_003256475.1	783	Toronto, Canada
CF170.7a	CF	2b	GCF_003256525.1	783	Toronto, Canada
CF170.7b	CF	2b	GCF_003257165.1	783	Toronto, Canada
CF170.7c	CF	2b	GCF_003256515.1	783	Toronto, Canada
CF170.7d	CF	2b	GCF_003257155.1	783	Toronto, Canada
CF170.7e	CF	2b	GCF_003256365.1	783	Toronto, Canada
CF170.7f	CF	2b	GCF_003256395.1	783	Toronto, Canada
CF170.7g	CF	2b	GCF_003257865.1	783	Toronto, Canada
CF170.7h	CF	2b	GCF_003256485.1	783	Toronto, Canada
CF170.7i	CF	2b	GCF_003257175.1	783	Toronto, Canada
CF170.7j	CF	2b	GCF_003257885.1	783	Toronto, Canada
CF170.8a	CF	2b	GCF_003256545.1	783	Toronto, Canada
CF170.8b	CF	2b	GCA_003256555.1	783	Toronto, Canada
CF170.8c	CF	2b	GCF_003257915.1	783	Toronto, Canada
CF170.8d	CF	2b	GCF_003257935.1	783	Toronto, Canada
CF170.8e	CF	2b	GCF_003257955.1	783	Toronto, Canada
CF170.8f	CF	2b	GCF_003257205.1	783	Toronto, Canada
CF170.8g	CF	2b	GCF_003257235.1	783	Toronto, Canada
CF170.8h	CF	2b	GCF_003256565.1	783	Toronto, Canada
CF170.8i	CF	2b	GCF_003257255.1	783	Toronto, Canada
CF170.8j	CF	2b	GCF_003257275.1	783	Toronto, Canada
CF170.9a	CF	2b	GCF_003256605.1	783	Toronto, Canada
CF170.9b	CF	2b	GCF_003257975.1	783	Toronto, Canada
CF170.9c	CF	2b	GCF_003257985.1	783	Toronto, Canada
CF170.9d	CF	2b	GCF_003257995.1	783	Toronto, Canada
CF170.9e	CF	2b	GCF_003258005.1	783	Toronto, Canada
CF170.9f	CF	2b	GCF_003257295.1	783	Toronto, Canada
CF170.9g	CF	2b	GCF_003256615.1	783	Toronto, Canada
CF170.9h	CF	2b	GCF_003258055.1	783	Toronto, Canada
CF170.9i	CF	2b	GCF_003258075.1	783	Toronto, Canada
CF170.9j	CF	2b	GCF_003256655.1	783	Toronto, Canada
CF2	CF	2a	GCF_000286575.1	1079	Unknown
CGD1	CGD	2b	GCF_000182255.1	1762	Maryland, USA
CGD2	CGD	1	GCF_000182275.1	442	Maryland, USA
CGD2M	CGD	1	GCF_000182295.1	442	Maryland, USA
D2095	CF	2b	GCF_000807825.1	16	Vancouver, Canada
D2214	CF	2b	GCF_000807815.1	16	Vancouver, Canada
DDS 15A-1	ENV	2a	GCF_000756005.1	802	Australia
DSOPR54	ENV	2a	GCA_002222845.1	1325	Singapore
DSOPR57	ENV	2a	GCA_002222875.1	ND	Singapore

DWS_42B-1	ENV	2a	GCF_000756965.1	809		Unknown
FDAARGOS_246	CF	1	GCF_003019965.1	650		Unknown
FDAARGOS_546	CLIN	1	GCF_003938705.1	355		Unknown
FDAARGOS_547	CLIN	2a	GCF_003812365.1	1809		USA
FDAARGOS_548	CLIN	2a	GCF_003812585.1	603		Unknown
HI3534	ENVH	1	GCF_001528605.1	620		USA
MSMB1128WGS	ENV	2b	GCF_001529795.1	1763		Australia
MSMB1272WGS	ENV	2a	GCF_001529925.1	1088		Australia
MSMB1535WGS	ENV	2b	GCF_001530485.1	1764		Australia
MSMB1640WGS	ENV	2a	GCF_001718995.1	802		Australia
MSMB1641WGS	ENV	2a	GCF_001531425.1	802		Australia
MSMB1794WGS	ENV	2b	GCF_001526715.1	1765		Australia
MSMB1916WGS	ENV	2a	GCF_001530985.1	1766		Australia
MSMB2008WGS	ENV	2a	GCF_001528045.1	1767		Australia
MSMB2021WGS	ENV	2a	GCF_001528425.1	1768		Australia
MSMB575WGS	ENV	2a	GCF_001534105.1	1769		Australia
MSMB576WGS	ENV	2a	GCF_001531955.1	1769		Australia
MSMB612WGS	ENV	2a	GCF_001532145.1	1770		Australia
NCTC13007	CF	1	GCF_900446205.1	650	LMG 13010	Belgium
NKI379	ENV	2a	GCF_001302465.1	1771		Taiwan
R-20526	ENV	1	GCF_001267755.1	836		Belgium

Table S2 Quality statistics of the 73 *B. multivorans* draft bacterial genomes sequenced in this study

Strain	Size	No. of contigs	GC content (%)	Shortest contig size	Median sequence size	Mean sequence size	Longest contig size	N50 Value
BCC0006	6272301	107	67.3	119	1564	58619.6	923050	239280
BCC0009	6342332	83	67.2	130	2945	76413.6	1042576	226583
BCC0032	6506850	70	67.1	129	1739	92955	597776	396722
BCC0033	6697341	82	67.1	101	1488	81674.9	873117	361065
BCC0043	6544285	95	67.1	121	1132	68887.2	946841	383582
BCC0047	7115961	80	66.6	122	2624	88949.5	1060012	360758
BCC0065	6507164	64	67.4	101	1505	101674.4	731639	407234
BCC0066	6510316	101	67.2	100	1564	64458.6	471548	225974
BCC0068	6440630	197	67.1	101	1519	32693.6	377625	154108
BCC0074	6541253	52	67.3	117	28069	125793.3	741944	401179
BCC0075	6612635	78	67.2	103	11573	84777.4	938953	268072
BCC0079	6991234	82	66.8	108	2155	85259	947587	337014
BCC0080	6706461	113	66.8	101	1294	59349.2	686101	289154
BCC0082	6621312	305	67.2	101	954	21709.2	304664	92055
BCC0084	6599540	137	67.1	101	1857	48171.8	377138	165313
BCC0087	6452471	62	67.4	105	4359	104072.1	859151	493463
BCC0093	6278118	46	67.23	312	40751	143203.1	739761	275222
BCC0096	6375796	91	67.2	109	2853	70063.7	1058392	233122
BCC0101	6384309	113	67.1	120	2162	56498.3	665106	219505
BCC0134	6414252	97	67.4	129	1574	66217.5	460055	308606
BCC0141	6171198	83	67.4	110	16803	74351.8	689872	216985
BCC0181	6537738	62	67.3	101	893	105554.1	835514	408687
BCC0188	6652537	144	67	114	9369	46198.2	572214	164596
BCC0225	6773647	59	67.1	127	1882	114807.6	1416856	6405560
BCC0241	6655513	94	67.1	125	7260	70803.3	631291	200202
BCC0246	6471839	65	67.3	122	16255	99566.8	678041	271451
BCC0247	6531380	92	67.3	111	2137	70993.3	731592	286611
BCC0255	6734567	87	67.2	108	1413	77497.6	510982	327645
BCC0264	6629658	60	67.1	114	27268	110494.3	519753	365645
BCC0266	6527276	92	67.3	115	12924	70948.7	447105	196973
BCC0292	6360854	110	67.1	103	1675	57904.6	771986	207640
BCC0293	6508922	198	67	102	1540	32956.4	537897	193063
BCC0300	6582551	74	67.2	108	1428	89061.5	729256	386587
BCC0303	6252321	96	67.2	18	8940	63799.2	707653	192319
BCC0317	6355099	81	67.5	106	9257	78458	758055	235405
BCC0321	6244833	106	67.32	102	2970	58975.8	659695	210819
BCC0375	6289743	100	67.3	103	3795	62897.4	893830	253362
BCC0381	6248359	82	67.3	134	3188	76199.5	725497	229512
BCC0384	6378847	159	67.4	100	449	40118.5	485421	244802
BCC0470	6984344	91	66.8	108	1413	76838.7	947619	337014
BCC0493	6530376	126	67.1	109	722	51828.4	450290	261403
BCC0497	6789721	97	66.9	121	2101	69997.1	592166	259787
BCC0533	6410978	78	67.35	124	1058	82311.4	1043364	421975
BCC0553	6694432	66	67.09	101	1625	101527.8	930655	405205
BCC0583	7040902	286	66.73	101	2597	24693.6	426567	131205
BCC0585	6447635	61	67.4	105	4145	105765.1	694113	337699
BCC0702	6455034	110	67.1	108	3289	58682.1	515667	241164
BCC0704	6449118	105	67.15	108	6604	61471.5	463159	217445
BCC0710	6234591	89	67.5	100	887	70051.6	684558	310618
BCC0729	6316285	139	67.31	105	1312	45512.6	468355	188298
BCC0737	6223066	76	67.3	102	18360	81882.4	599007	239689
BCC0814	6273978	110	67.3	101	2755	57036.2	427585	238427
BCC0865	6791523	134	66.6	106	2148	50683	557856	262358
BCC0901	6497146	61	67.17	13	1752	106579.9	866533	529217
BCC0904	6029426	163	67.4	100	5437	36990.3	380474	106352
BCC0907	6658191	99	66.93	124	1949	67349.9	839825	265179
BCC0915	6277422	87	67.32	121	2755	72227.8	594460	243519
BCC0921	6786237	158	66.6	106	2274	42950.9	557856	197853
BCC0962	6277315	88	67.32	121	2972	71405.8	594459	243519

BCC0968	6785185	143	66.58	107	2303	47500.3	557857	231462
BCC1147	6576406	109	67.1	105	5295	60334	565382	173107
BCC1148	6573791	130	67.1	104	1652	50567.6	365455	171919
BCC1177	6520943	160	67.1	101	2151	40755.9	470646	148928
BCC1185	6383426	65	67.4	105	4145	98206.6	692368	368878
BCC1190	6366933	112	67.1	109	6177	56847.6	378394	176650
BCC1271	6997886	219	66.8	101	2320	32022.5	434582	151267
BCC1272	6882314	163	66.8	117	3934	42222.8	772166	178859
BCC1367	6246910	99	67.2	108	7439	63156.2	707677	207005
BCC1368	6273724	109	67.2	102	2542	57557.1	796635	218602
BCC1384	6246946	97	67.2	108	7439	64458.8	707668	220614
BCC1385	6606699	112	66.9	101	1752	58988.4	544646	236600
BCC1421	6609469	70	67.2	102	14809	94461.5	940166	352409
**Average**	**6514719.4**	**107.09722**	**67.143056**	**109.81944**	**5156.1111**	**69942.958**	**676024.39**	**348751.403**

Table S3. PCR primer sequences designed for all four *B. multivorans* lineage-specific target genes

Target Gene	Primer Name	Primer Sequence (5' to 3') [c]	Primer Length (bp)	Position	Annealing Temperature (°C)	Product Size (bp)
YiaJ_1	YIAJBM1F	**AT**C**C**GG**C**AA**CT**ATTCGCT	18	4007519-4007536[a]	53.3	537
	YIAJBM1R	**CAACGCTTTCCGTAGATG**	18	4007000-4007017[a]		
ghrB_1	GHRBBM1F	CAAGCAACCGACCGAA**AG**	18	4008677-4008694[a]	53.0	744
	GHRBBM1R	GGAGACAG**A**ATCACGTT**C**	18	4009403-4009420[a]		
naiP_3	NAIPBM1F	**A**GCCGC**C**GA**AC**AA**A**G**A**TT**GA**	20	4014235-4014254[a]	55.7	981
	NAIPBM1R	CTGAAGCCGGTCAGAAA**G**	18	4015198-4015215[a]		
glnM_2	GLNMBM2F	T**G**AA**T**G**CCG**GCCACGTA**TG**	19	1792198-1792216[b]	55.5	322
	GLNMBM2R	GACGCATACGACAG**T**TCC	18	1791895-1791912[b]		

[a] Position relative to the BCC0084 (lineage 1) complete genome

[b] Position relative to the ATCC 17616 (lineage 2a) complete genome

[c] Mismatches for each primer sequence are highlighted in bold. Red indicates a mismatch in all strains of the opposing lineage and blue indicates mismatches within target lineage strains

Table S4. Growth kinetics of the *B. multivorans* strains showing growth rate, lag phase and maximum OD

Isolate	Lineage	Statistical Model	Growth Rate (h^{-1})	Lag Phase (λ)	Maximum OD
**Lineage 1**					
BCC0006	1	richards	0.02309	5.307211	0.359725
BCC0009	1	gompertz	0.044397	2.467438	0.375609
BCC0080	1	richards	0.037799	4.237065	0.397279
BCC0084	1	richards	0.033811	3.66836	0.382379
BCC0101	1	richards	0.048303	3.296633	0.380578
BCC0141	1	richards	0.020178	4.394878	0.305782
BCC0303	1	richards	0.020057	11.59027	0.351278
BCC0375	1	richards	0.019472	3.500513	0.36421
BCC0702	1	richards	0.016862	1.362989	0.335749
BCC0737	1	richards	0.031135	7.56969	0.373028
BCC0814	1	gompertz	0.016017	2.446835	0.394281
BCC0865	1	richards	0.018893	7.541105	0.341761
BCC0904	1	richards	0.04133	3.591944	0.394376
BCC0921	1	richards	0.020943	12.20387	0.333387
BCC1177	1	richards	0.020631	4.565362	0.337437
BCC1190	1	richards	0.040893	2.944074	0.371125
BCC1385	1	richards	0.044277	2.76252	0.362452
Average (SE)	-	-	0.029 (0.003)	4.908 (0.752)	0.362 (0.006)
**Lineage 2a**					
BCC0047	2a	richards	0.02602	4.995805	0.394584
BCC0066	2a	logistic	0.045924	4.124916	0.37575
BCC0074	2a	richards	0.053576	4.486977	0.358003
BCC0188	2a	richards	0.018039	1.466231	0.357657
BCC0225	2a	gompertz	0.018589	2.508097	0.326467
BCC0264	2a	logistic	0.040676	3.827443	0.373854
BCC0266	2a	richards	0.037115	3.610816	0.39047
BCC0317	2a	gompertz	0.042476	2.43051	0.380466
BCC1272	2a	richards	0.059492	4.869471	0.327952
Average (SE)	-	-	0.038 (0.005)	3.591 (0.405)	0.365 (0.008)
**Lineage 2b**					
BCC0033	2b	logistic	0.035193	4.217101	0.369231
BCC0043	2b	richards	0.024118	4.811215	0.373581
BCC0065	2b	logistic	0.037451	4.248356	0.369135
BCC0068	2b	richards	0.014588	7.214126	0.331623
BCC0075	2b	richards	0.016353	4.902274	0.312914
BCC0079	2b	richards	0.029847	3.006941	0.366436
BCC0082	2b	richards	0.03056	3.437512	0.372343
BCC0087	2b	richards	0.042158	3.400263	0.38021
BCC0096	2b	richards	0.041078	3.280156	0.37302
BCC0241	2b	logistic	0.04607	3.650837	0.372825
BCC0246	2b	gompertz	0.043649	3.473084	0.411566
BCC0247	2b	richards	0.014531	0.7697	0.373392
BCC0269	2b	richards	0.021785	10.1249	0.327047
BCC0384	2b	gompertz.exp	0.043328	3.879821	0.355711
BCC0493	2b	richards	0.02316	12.34905	0.368741
BCC0497	2b	richards	0.016688	7.899568	0.35352
BCC0710	2b	richards	0.035296	4.439582	0.349044
BCC1147	2b	gompertz.exp	0.04965	4.450516	0.335572
BCC1148	2b	richards	0.034645	5.522386	0.401968
BCC1185	2b	richards	0.020373	9.216607	0.319333
Average (SE)	-	-	0.031 (0.003)	5.214 (0.624)	0.361 (0.006)
**Other**					
BCC0302	NA	gompertz	0.045548	3.481668	0.383461
BCC1368	Other	richards	0.028289	3.644625	0.364084

Table S5. Swimming and swarming motility within the *B. multivorans* strains

Strain	Mean motility zone (mm) within each growth medium and motility phenotype [b,c]		
	**0.3% LB (Swimming)**	**0.5% LB (Swarming)**	**0.5% BSM-G (Swarming)**
ATCC 17616 [a]	21.8	7.8	22.2
BCC0006	10.3	0.0	0.0
BCC0009	57.8	14.4	90.0
BCC0032	18.8	2.6	3.8
BCC0033	25.2	12.0	9.8
BCC0043	15.3	2.8	0.0
BCC0047	25.3	10.0	30.1
BCC0065	24.1	11.8	8.8
BCC0066	22.3	10.4	ND
BCC0068	0.0	0.0	0.0
BCC0074	20.1	3.8	3.1
BCC0075	41.2	17.3	77.3
BCC0079	29.4	18.8	ND
BCC0080	26.8	3.5	0.0
BCC0082	31.8	9.9	ND
BCC0084	58.6	24.3	36.3
BCC0087	27.3	17.4	24.6
BCC0096	38.8	12.3	18.1
BCC0101	23.7	5.3	8.8
BCC0141	13.9	8.3	13.6
BCC0188	ND	ND	ND
BCC0225	30.1	11.7	ND
BCC0241	29.5	16.4	18.0
BCC0246	18.7	5.4	5.8
BCC0247	13.8	5.3	6.5
BCC0264	21.3	4.7	3.5
BCC0266	25.1	10	11.1
BCC0269	8.5	5.4	4.0
BCC0302	46.1	14.3	ND
BCC0303	2.3	2.7	14.8
BCC0317	90.0	19.5	87.5
BCC0375	19.8	11.8	9.0
BCC0381	ND	ND	ND
BCC0384	17.3	5.1	10.8
BCC0493	6.8	4.5	0.0
BCC0497	5.3	2.8	16.2
BCC0702	47.1	32.6	69.1
BCC0710	22.3	8	6.3
BCC0737	9.9	3.8	11.8
BCC0814	24.4	14.3	16.8
BCC0865	7.8	5.7	4.3
BCC0904	3.2	2	0.0
BCC0921	10.7	7.5	3.3
BCC1147	24.8	8.3	26.6
BCC1148	14.7	5.3	14.3
BCC1177	60.9	24.6	90.0
BCC1185	17.3	9.5	1.2
BCC1190	59.6	17.8	90.0
BCC1272	25.8	7.3	70.7
BCC1368	7.7	ND	ND

BCC1385	25.6	14.3	40.4

[a] *B. multivorans* ATCC 17616 was used as a positive motile control for all assays
[b] Highly motile strains (≤50 mm average motility diameter) are highlighted in blue
[c] ND = mean not determined due to variable results

Table S6. Colony morphology and EPS production of *B. multivorans*

Lineage	Strain	Morphology				
		Shape	Size	Appearance	Congo Red Agar	EPS production (YEM agar)
1	BCC0006	Round	Small	Shiny	Red	-
	BCC0009	Irregular	Medium	Shiny	Pink to pink with orange centre	++++
	BCC0010	Round	Small	Shiny	Pink with red centre	++++
	BCC0067	Round	Small	Shiny	Pink with red centre	++++
	BCC0080	Round	Medium	Shiny	Pink with red centre	++
	BCC0084	Irregular	Medium	Shiny	Pink with orange centre	+++
	BCC0093	Round	Small	Shiny	Pink with red centre	++++
	BCC0101	Round	Medium	Shiny	Pink with red centre	++
	BCC0115	Irregular	Medium	Shiny	Pink with orange centre	++++
	BCC0141	Round	Small	Shiny	pink with red centre	++++
	BCC0149	Round	Small	Shiny	Pink with orange-red centre	++++
	BCC0175	Round	Medium	Shiny	Pink with orange-red centre	++
	BCC0292	Round	Medium	Shiny	Pink with red centre	++++
	BCC0293	Round	Small	Shiny	Pink with orange centre	++
	BCC0303	Round	Small	Shiny	Pink	+++
	BCC0321	Round	Small	Shiny	Pink	+
	BCC0375	Round	Medium	Shiny	Pink with red centre	++
	BCC0702	Round	Small	Shiny	Pink with orange centre	+++
	BCC0704	Round	Small	Shiny	Pink with orange centre	+++
	BCC0737	Round	Small	Shiny	Pink with red centre	+++
	BCC0814	Round	Medium	Shiny	Pink to pink with orange centre	+++
	BCC0865	Round	Small	Shiny	Pink with red centre	++
	BCC0904	Round	Small	Shiny	Pink	+
	BCC0915	Round	Small	Shiny	Pink with orange-red centre	+++
	BCC0921	Round	Small	Shiny	Pink with orange centre	+
	BCC0962	Round	Small	Shiny	Pink with red centre	++++
	BCC0968	Round	Small	Shiny	Pink with orange-red centre	+++
	BCC1177	Round	Small	Shiny	Pink with orange centre	+++
	BCC1190	Round	Small	Shiny	Pink with red centre	++++
	BCC1367	Round	Small	Shiny	Orange to red	++
	BCC1384	Round	Small	Shiny	Pink	++++
	BCC1385	Irregular	Medium	Shiny	Pink with red centre	++++
2	ATCC17616	Irregular	Medium	Shiny	Pink with red centre	+++
	BCC0005	Round	Medium	Shiny	Pink with red centre	++++
	BCC0008	Round	Small	Shiny	Pink with orange-red centre	++++
	BCC0031	Round	Medium	Shiny	Pink with orange-red centre	++++
	BCC0032	Round	Medium	Shiny	Pink with red centre	++
	BCC0033	Irregular	Medium	Shiny	Pink with red centre	+++
	BCC0037	Round	Medium	Shiny	Pink with orange-red centre	++++
	BCC0043	Round	Small	Shiny	Pink with orange centre	++
	BCC0047	Round	Medium	Shiny	pink	++++
	BCC0050	Round	Medium	Shiny	Pink with orange-red centre	++++
	BCC0059	Round	Small	Shiny	Orange to red	+++
	BCC0065	Round	Medium	Shiny	Pink to pink with orange centre	++
	BCC0066	Round	Medium	Shiny	Pink with red centre	++
	BCC0068	Round	Small	Shiny	pink	-
	BCC0074	Round	Medium	Shiny	Pink with red centre	++
	BCC0075	Round	Small	Shiny	Pink with orange centre	++++
	BCC0079	Round	Medium	Shiny	Pink	++++
	BCC0082	Irregular	Medium	Shiny	Pink with red centre	++
	BCC0087	Round	Medium	Shiny	pink with orange centre	++
	BCC0089	Round	Medium	Shiny	Pink with orange-red centre	++++
	BCC0096	Round	Medium	Shiny	Pink with orange centre	+++
	BCC0099	Round	Medium	Shiny	Pink with red centre	+++
	BCC0102	Round	Small	Shiny	Pink with red centre	++++
	BCC0134	Round	Small	Shiny	Pink with red centre	++
	BCC0181	Round	Small	Shiny	Pink with red centre	++++
	BCC0188	Round	Small	Shiny	Pink with red centre	-
	BCC0225	Irregular	Medium	Shiny	Red	+++
	BCC0241	Round	Medium	Shiny	pink with red centre	++
	BCC0244	Round	Medium	Shiny	Pink with red centre	+
	BCC0246	Round	Medium	Shiny	Pink with red centre	++++
	BCC0247	Round	Medium	Shiny	Pink with red centre	++
	BCC0255	Round	Small	Shiny	Pink with orange centre	++++
	BCC0264	Round	Medium	Shiny	Pink with red centre	+
	BCC0266	Round	Medium	Shiny	pink with red centre	++
	BCC0269	Round	Medium	Shiny	pink with red centre	+++
	BCC0300	Round	Small	Shiny	Pink with orange centre	+++
	BCC0317	Irregular	Medium	Shiny	Pink with orange centre	++++
	BCC0384	Round	Small	Shiny	pink	+
	BCC0470	Round	Medium	Shiny	Pink with red centre	+++
	BCC0493	Round	Small	Shiny	Pink	-
	BCC0497	Round	Small	Shiny	Pink	-

	BCC0533	Irregular	Medium	Shiny	Pink with red centre	++++
	BCC0553	Round	Small	Shiny	Pink with orange-red centre	+
	BCC0583	Round	Small	Shiny	Pink with red centre	++
	BCC0585	Round	Small	Shiny	Pink with red centre	+++
	BCC0585	Round	Small	Shiny	Pink with orange-red centre	+++
	BCC0710	Round	Small	Shiny	Pink with red centre	++
	BCC0729	Irregular	Medium	Shiny	Pink with red centre	+++
	BCC0901	Round	Small	Shiny	Red	+++
	BCC0907	Round	Medium	Shiny	Pink with red centre	+++
	BCC1147	Round	Small	Shiny	Pink with orange centre	++
	BCC1148	Round	Small	Shiny	pink	++
	BCC1185	Round	Small	Shiny	Pink	+++
	BCC1271	Round	Small	Shiny	Pink with orange centre	+++
	BCC1272	Round	Small	Shiny	Pink with red centre	+++
	BCC1421	Round	Small	Shiny	Pink with orange-red centre	++++
Other	BCC1368	Round	Small	Shiny	Red	+

Supplementary Figures

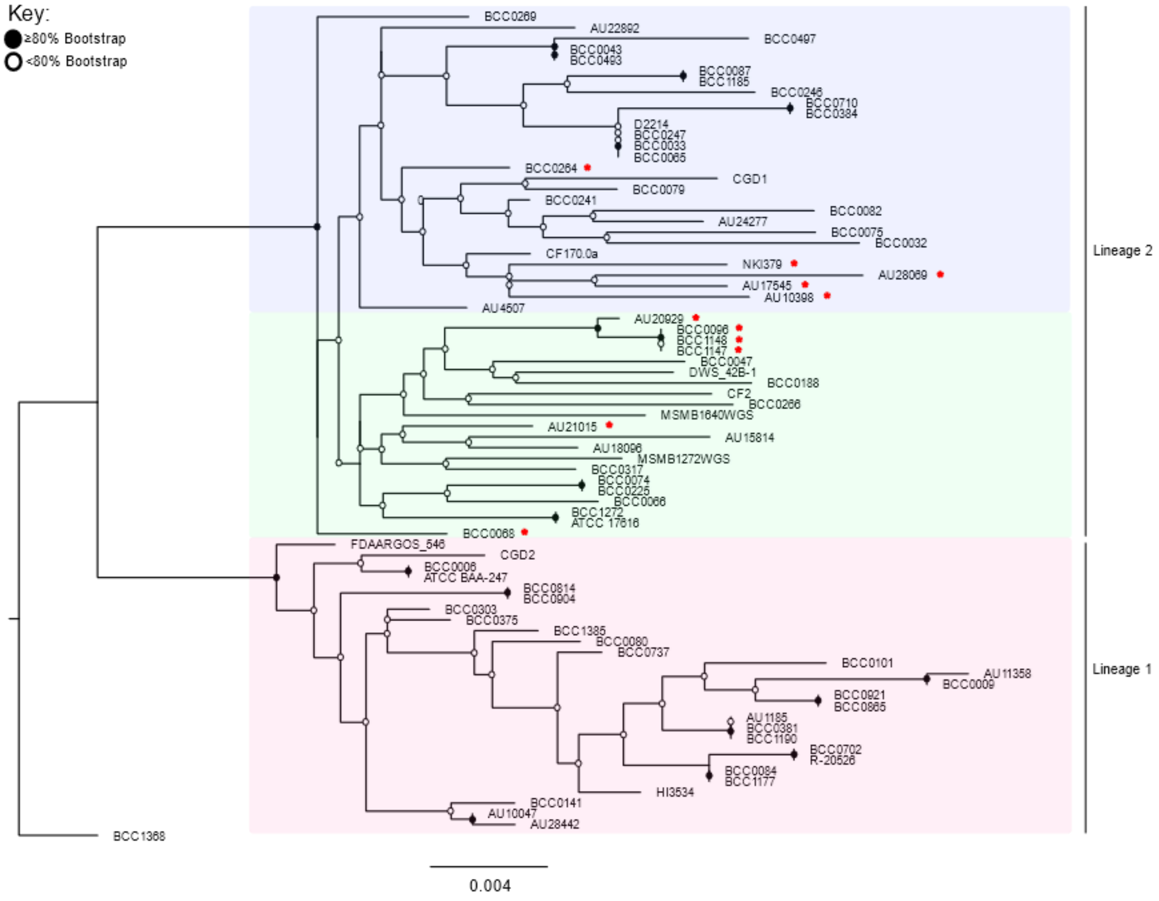

Figure S1. MLST-gene phylogeny of the 77 genome B. multivorans strain panel. The RAxML (100 bootstraps) phylogenetic tree was created by extracting the MLST sequences from the sequenced whole genomes using MLST check (Page et al. 2016b). Bootstrap values are indicated using the key. Filled circles represent a bootstrap of ≥80% and a hollow (white) circle means confidence of ≤80%. Lineages are shown on the right hand side of the figure, with colour coding illustrating the lineages and sub-lineages. Lineage 1 = red, lineage 2a = green, lineage 2b = blue. The red asterix next to strain names represents the flipped genomes in lineage 2. Scale bar represents the phylogenetic distance of 0.004 nucleotide substitutions.

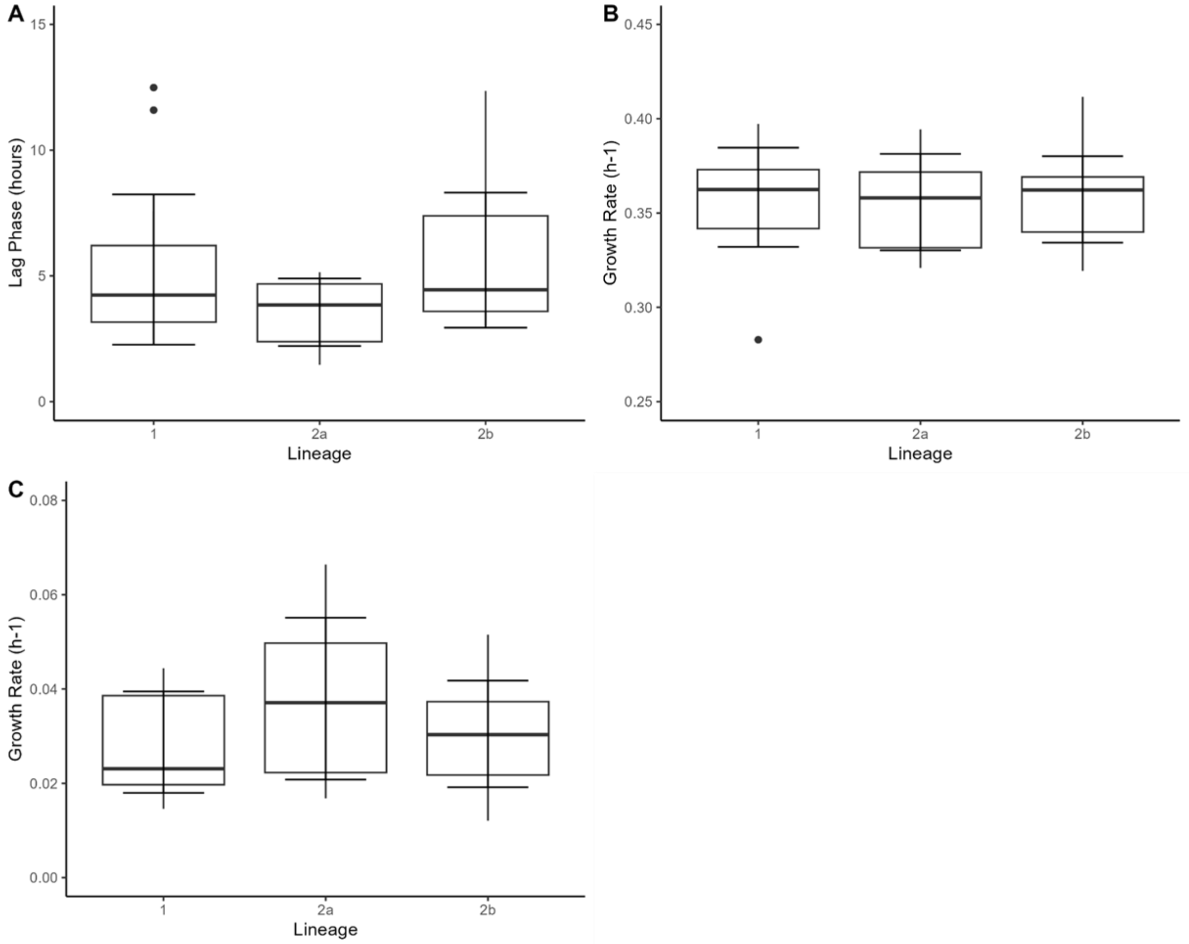

Figure S2. Growth parameters of the *B. multivorans* strains (n = 50) at 48 hours. The box plots have been drawn from data produced in R using the GroFit package: (A) growth rate (h^{-1}), (B) lag phase (hours), and (C) maximum growth (OD 480-520 nm). All box plots are annotated with the model strains on the right-hand side (red) and display the upper quartile, mean, and lower quartile. Outliers have also been added to the plots. Each parameter has been compared by *B.* multivorans lineage and no statistically significant *B. multivorans* lineage-specific differences in these growth parameters were identified ($p > 0.05$ for comparison of mean for all plots).

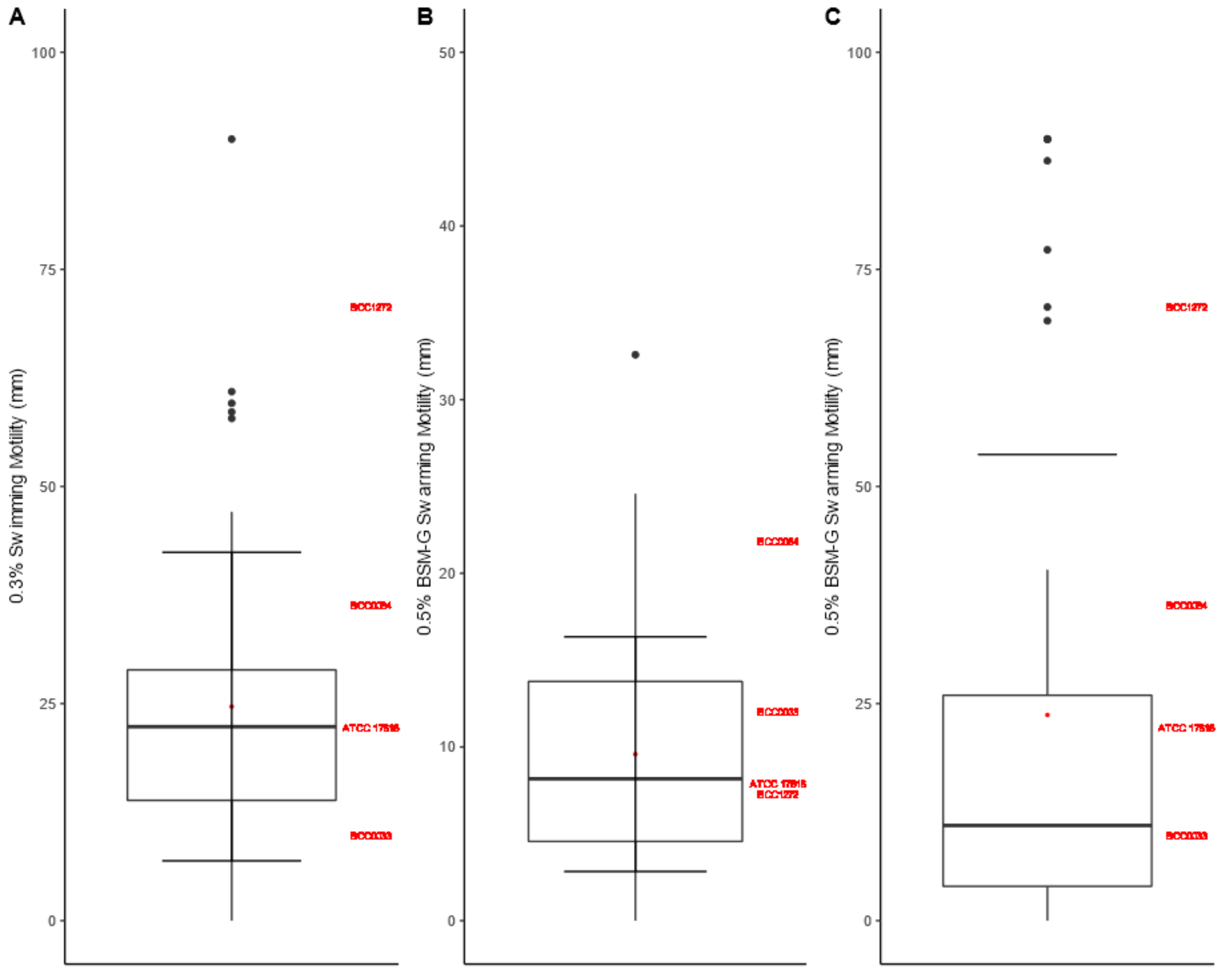

Figure S3. Swimming and swarming motility in the *B. multivorans* strain panel after 24-hours incubation at 37°C. (A) Swimming motility was performed on 0.3% LB agar (n = 49). Swarming motility was performed on (B) 0.5% LB agar (n = 48) and (C) 0.5% BSM-G agar (n = 44). Model *B. multivorans* strains (n = 4) are annotated in red on the righthand side of each box plot. No motility associations statistically linked to each *B. multivorans* lineage were identified.

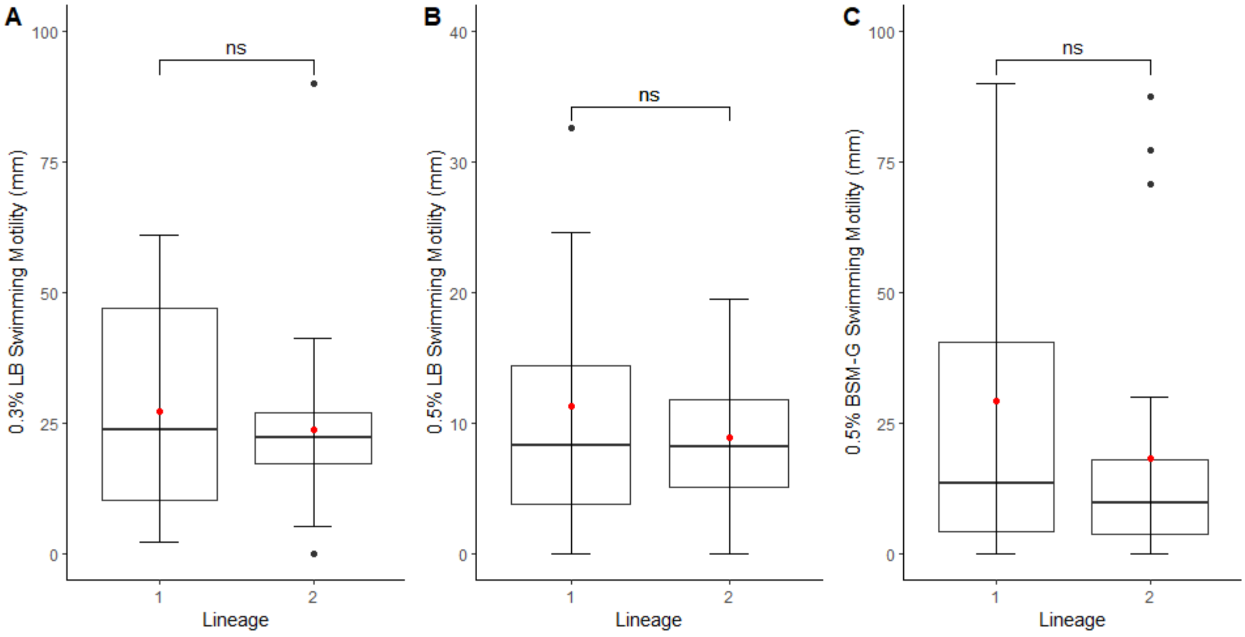

Figure S4. Comparison of motility between lineage 1 and lineage 2 *B. multivorans* strains. The box plots show: (A) 0.3% LB swimming motility: Lineage 1 (n = 17) and lineage 2 (n = 30); (B) 0.5% LB swarming motility: Lineage 1 (n = 17) and lineage 2 (n = 30); (C) 0.5% BSM-G swarming motility: Lineage 1 (n = 17) and lineage 2 (n = 25). Statistical analysis was performed in R using a non-parametric Kruskal-Wallis test was performed on all motilities. No significant differences in motility were observed at p = 0.05 as indicated (ns).

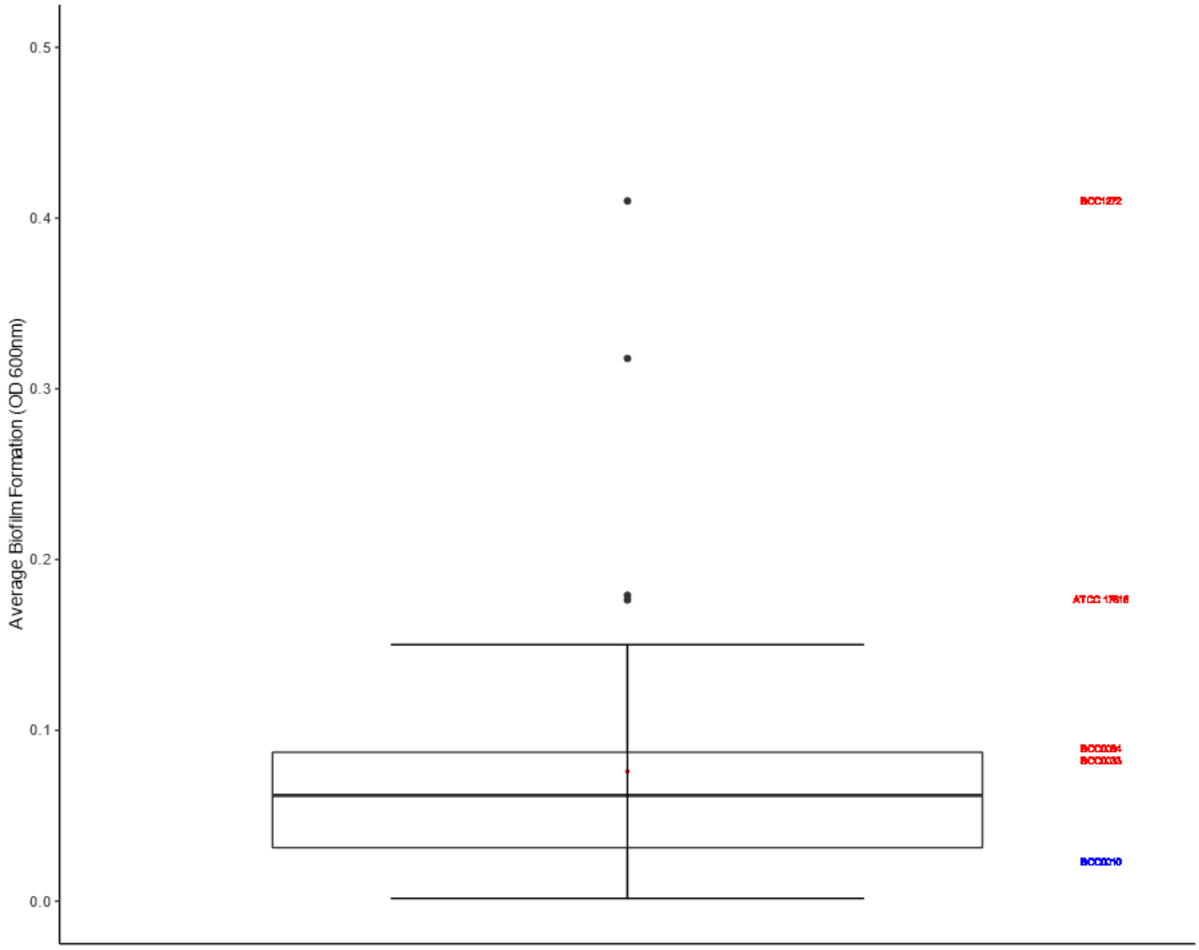

Figure S5. Biofilm formation of the *B. multivorans* strain panel (n = 49) after 24-hours. Among of biofilm was assessed using the crystal violet assay, reading the results using a plate reader at 600_{nm}. Biofilm controls were *B. multivorans* ATCC 17616 for the 'high' former and BCC0010 (blue) for the 'low' control. Model CF B. multivorans strains are noted in red on the right-hand side of the box plot. Box plots show the mean, lower quartile, and upper quartile for biofilm staining. Outliers are also shown. No biofilm formation abilities statistically linked to each *B. multivorans* lineage were identified.

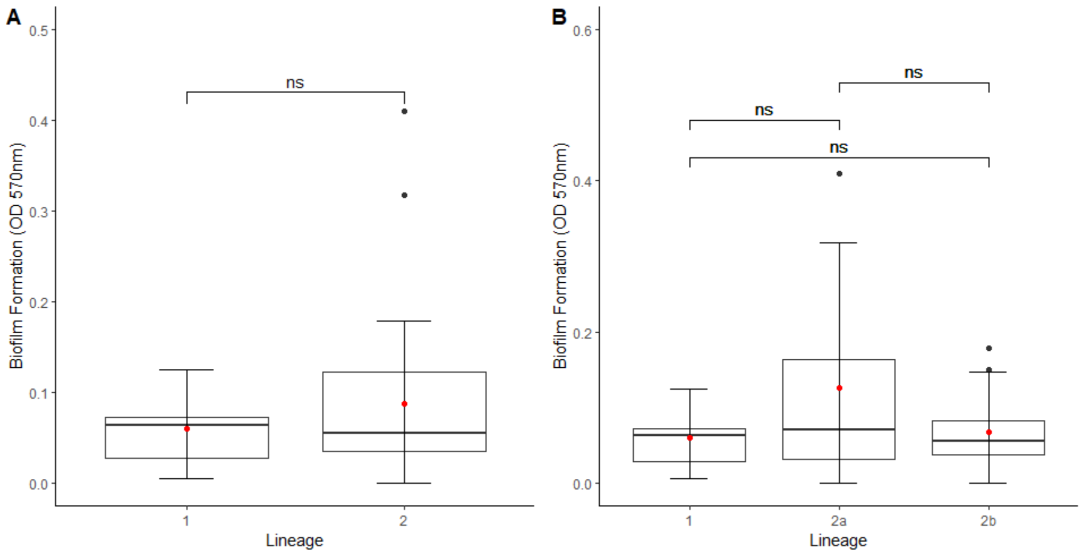

Figure S6. Comparison of biofilm formation between the *B. multivorans* lineages. *B. multivorans* ATCC 17616 was used as a high biofilm control and included in all the lineage comparisons made using the *B. multivorans* sub-panel (n=49 strains compared). The box plots show: (A) Comparison of biofilm formation between lineage 1 (n = 17) and lineage 2 (n = 30); (B) Comparison of biofilm formation between lineage 1 (n = 17), lineage 2a (n = 11) and lineage 2b (n = 19). Statistical analysis was performed in R using a non-parametric Kruskal-Wallis test for two comparisons or Dunn-Test and Benjamini-Hochberg test for three comparisons. No significance differences in biofilm formation were observed at p = 0.05 as indicated (ns).
